# 3-Benzyl-7-(2,4-dichloro­phen­yl)-4*H*-1,3,4-thia­diazolo[2,3-*c*][1,2,4]triazin-4-one

**DOI:** 10.1107/S1600536812021253

**Published:** 2012-05-16

**Authors:** Hoong-Kun Fun, Suhana Arshad, B. K. Sarojini, U. A. Imran, B.G. Krishna

**Affiliations:** a X-ray Crystallography Unit, School of Physics, Universiti Sains Malaysia, 11800 USM, Penang, Malaysia; b Department of Chemistry, P. A. College of Engineering, Mangalore 574 153, India

## Abstract

In the title compound, C_17_H_10_Cl_2_N_4_OS, the phenyl ring and the H atoms attached to the adjacent C atom are disordered over two positions, with refined site occupancies of 0.509 (8) and 0.491 (8). The planar 4*H*-1,3,4-thia­diazolo[2,3-*c*][1,2,4]triazine ring system [maximum deviation = 0.048 (3) Å] forms dihedral angles of 76.9 (5), 74.9 (5) and 9.88 (12)°, respectively, with the major and minor parts of the disordered phenyl ring and with the dichloro-substituted benzene ring. In the crystal, pairs of C—H⋯O hydrogen bonds link the mol­ecules, forming inversion dimers with an *R*_2_^2^(18) graph-set motif. A short S⋯N contact of 2.801 (3) Å is observed between the dimers.

## Related literature

For applications of thia­diazole derivatives, see: Kurtzer (1965 ▶); Sandstrom (1968 ▶); Eue & Tietz (1970 ▶); Holla *et al.* (1988 ▶, 1998 ▶). For a related structure, see: Zhang *et al.* (2011 ▶); Fun *et al.* (2011 ▶); Ma & Yang (2008 ▶); Yu *et al.* (2007 ▶); Jia *et al.* (2011 ▶). For the stability of the temperature controller used for data collection, see: Cosier & Glazer (1986 ▶). For hydrogen-bond motifs, see: Bernstein *et al.* (1995 ▶).
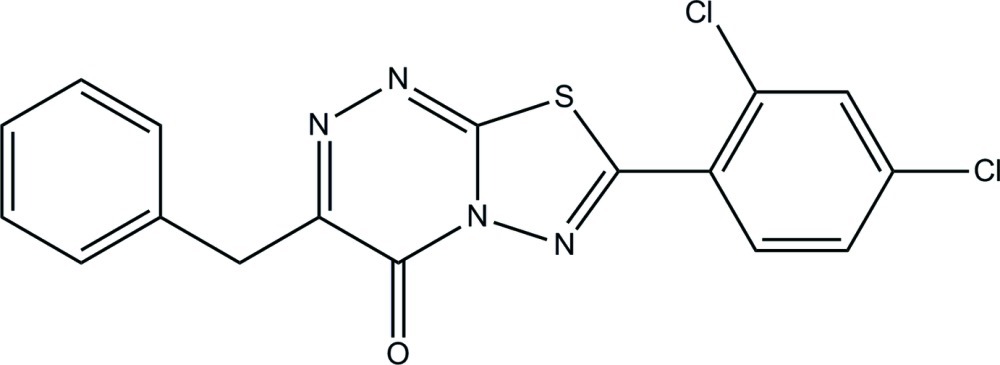


## Experimental

### 

#### Crystal data

C_17_H_10_Cl_2_N_4_OS
*M* *_r_* = 389.25
Triclinic, 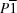

*a* = 4.4961 (1) Å
*b* = 13.4412 (5) Å
*c* = 14.4588 (5) Å
α = 70.620 (2)°
β = 85.956 (2)°
γ = 83.063 (2)°
*V* = 817.81 (5) Å^3^
*Z* = 2
Mo *K*α radiation
μ = 0.54 mm^−1^
*T* = 100 K
0.21 × 0.10 × 0.09 mm

#### Data collection

Bruker SMART APEXII CCD area-detector diffractometer
Absorption correction: multi-scan (*SADABS*; Bruker, 2009 ▶) *T*_min_ = 0.894, *T*_max_ = 0.955
8906 measured reflections
4086 independent reflections
2479 reflections with *I* > 2σ(*I*)
*R*_int_ = 0.053

#### Refinement

*R*[*F* ^2^ > 2σ(*F* ^2^)] = 0.060
*wR*(*F* ^2^) = 0.129
*S* = 1.04
4086 reflections
281 parameters
180 restraints
H-atom parameters constrained
Δρ_max_ = 0.42 e Å^−3^
Δρ_min_ = −0.38 e Å^−3^

### 

Data collection: *APEX2* (Bruker, 2009 ▶); cell refinement: *SAINT* (Bruker, 2009 ▶); data reduction: *SAINT*; program(s) used to solve structure: *SHELXTL* (Sheldrick, 2008 ▶); program(s) used to refine structure: *SHELXTL*; molecular graphics: *SHELXTL*; software used to prepare material for publication: *SHELXTL* and *PLATON* (Spek, 2009 ▶).

## Supplementary Material

Crystal structure: contains datablock(s) global, I. DOI: 10.1107/S1600536812021253/is5127sup1.cif

Structure factors: contains datablock(s) I. DOI: 10.1107/S1600536812021253/is5127Isup2.hkl

Supplementary material file. DOI: 10.1107/S1600536812021253/is5127Isup3.cml

Additional supplementary materials:  crystallographic information; 3D view; checkCIF report

## Figures and Tables

**Table 1:** Hydrogen-bond geometry (Å, °)

*D*—H⋯*A*	*D*—H	H⋯*A*	*D*⋯*A*	*D*—H⋯*A*
C2—H2*A*⋯O1^i^	0.95	2.36	3.226 (5)	151

Symmetry code: (i) 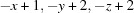.

**Fig. 1.**
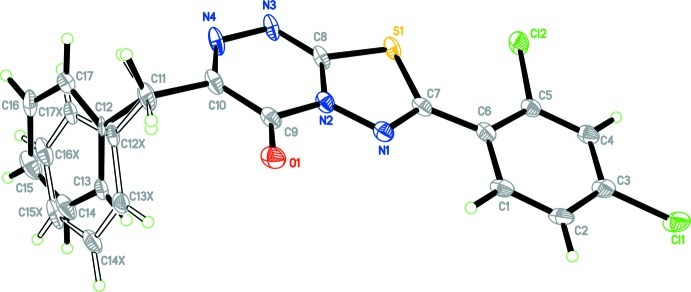
The molecular structure of the title compound, showing 30% probability displacement ellipsoids and the atom-numbering scheme. All disordered components are shown.

**Fig. 2.**
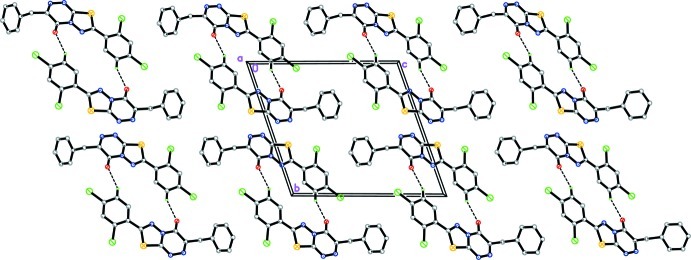
A packing diagram of the title compound viewed along the a axis. Dashed lines represent the intermolecular hydrogen bonds. Only major disordered component is shown.

## supplementary crystallographic information

### Comment

Several thiadiazoles find important application in the field of medicine, agriculture and industry (Kurtzer, 1965; Sandstrom, 1968). Thiadiazoles were also used as effective herbicides and fungicides (Eue & Tietz, 1970). The antibacterial activity of some of the thiadiazolotriazinone derivatives were reported (Holla *et al.*, 1998). The presence of substituents like aryloxymethyl and anilinomethyl on thiadiazole moiety were known to enhance the biological activities (Holla *et al.*, 1988). The crystal structures of some of the thiadiazole derivatives have been reported (Zhang *et al.*, 2011; Fun *et al.*, 2011; Ma & Yang, 2008). The present work describes the synthesis and crystal structure of the title compound (C_17_H_10_Cl_2_N_4_OS), 3-benzyl-7-(2,4-dichlorophenyl)-4*H* -[1,3,4]thiadiazolo[2,3-*c*][1,2,4]triazin-4-one. The compound was prepared by the reaction of 4-amino-6-benzyl-3-mercapto-1,2,4-triazin- 5(4*H*)-one with 2,4 dichlorobenzoic acid.

The molecular structure is shown in Fig. 1. The benzene ring and the hydrogen atoms which are attached to atom C11 are disordered over two positions with refined site-occupancies of 0.509 (8): 0.491 (8) ratio. Bond lengths and angles are within normal ranges. The 4*H*-[1,3,4]thiadiazolo [2,3-*c*][1,2,4]triazine ring (S1/N1/N2/N3/N4/C7–C10; maximum deviation = 0.048 (3) Å at atom C9) forms dihedral angles of 76.9 (5), 74.9 (5) and 9.88 (12)° with the major and minor parts of the disordered benzene ring (C12–C17 & C12X–C17X) and the dichloro-substituted benzene ring (C1–C6), respectively.

In the crystal packing (Fig. 2), two neighbouring molecules are linked by intermolecular C2—H2A···O1 hydrogen bonds (Table 1) into inversion dimers, forming R^2^_2_(18) graph-set motifs (Bernstein *et al.*, 1995). A short S1···N3 contact [2.801 (3) Å, symmetry code: -*x*,1 - *y*,2 - *z*] is observed between the dimers and comparable with those short contacts of the previously reported structures (Yu *et al.*, 2007; Jia *et al.*, 2011).

### Experimental

A mixture of 4-amino-6-benzyl-3-mercapto-1,2,4-triazin-5(4*H*)-one (2.34 g, 0.01 mol), 2,4-dichlorobenzoic acid (1.91 g, 0.01 mol) and phosphorus oxychloride (10 ml) was refluxed on a water bath for about 5 h. Excess of phosphorus oxychloride was removed under reduced pressure. The reaction mixture was cooled and poured onto crushed ice. The resulting solid product was filtered, washed with sodium bicarbonate solution (2%), followed by distilled water. It was dried and recrystallized from aqueous dioxane. The single crystals were grown by slow evaporation from a mixture of ethanol and dichloromethane. (1:1) (*M.p.* = 473–475 K).

### Refinement

The benzene ring and the hydrogen atoms which are attached to atom C11 are disordered over two positions with refined site-occupancies of 0.509 (8): 0.491 (8) ratio. All the H atoms were positioned geometrically (C—H = 0.95 and 0.99 Å) and refined using a riding model with *U*_iso_(H) = 1.2*U*_eq_(C). Similarity and rigid bond restraints were used in the final refinement. Two outliers (0 1 1) and (0 0 1) were omitted.

### Crystal data

**Table:**

C_17_H_10_Cl_2_N_4_OS	*Z* = 2
*M**_r_* = 389.25	*F*(000) = 396
Triclinic, *P*1	*D*_x_ = 1.581 Mg m^−^^3^
Hall symbol: -P 1	Mo *K*α radiation, λ = 0.71073 Å
*a* = 4.4961 (1) Å	Cell parameters from 5482 reflections
*b* = 13.4412 (5) Å	θ = 2.5–31.5°
*c* = 14.4588 (5) Å	µ = 0.54 mm^−^^1^
α = 70.620 (2)°	*T* = 100 K
β = 85.956 (2)°	Block, yellow
γ = 83.063 (2)°	0.21 × 0.10 × 0.09 mm
*V* = 817.81 (5) Å^3^	

### Data collection

**Table:**

Bruker SMART APEXII CCD area-detector diffractometer	4086 independent reflections
Radiation source: fine-focus sealed tube	2479 reflections with *I* > 2σ(*I*)
Graphite monochromator	*R*_int_ = 0.053
φ and ω scans	θ_max_ = 28.5°, θ_min_ = 2.5°
Absorption correction: multi-scan (*SADABS*; Bruker, 2009)	*h* = −6→6
*T*_min_ = 0.894, *T*_max_ = 0.955	*k* = −18→18
8906 measured reflections	*l* = −18→19

### Refinement

**Table:**

Refinement on *F*^2^	Primary atom site location: structure-invariant direct methods
Least-squares matrix: full	Secondary atom site location: difference Fourier map
*R*[*F*^2^ > 2σ(*F*^2^)] = 0.060	Hydrogen site location: inferred from neighbouring sites
*wR*(*F*^2^) = 0.129	H-atom parameters constrained
*S* = 1.04	*w* = 1/[σ^2^(*F*_o_^2^) + (0.0449*P*)^2^ + 0.2058*P*] where *P* = (*F*_o_^2^ + 2*F*_c_^2^)/3
4086 reflections	(Δ/σ)_max_ = 0.001
281 parameters	Δρ_max_ = 0.42 e Å^−^^3^
180 restraints	Δρ_min_ = −0.38 e Å^−^^3^

### Special details

**Table:**

Experimental. The crystal was placed in the cold stream of an Oxford Cryosystems Cobra open-flow nitrogen cryostat (Cosier & Glazer, 1986) operating at 100.0 (1) K.
Geometry. All e.s.d.'s (except the e.s.d. in the dihedral angle between two l.s. planes) are estimated using the full covariance matrix. The cell e.s.d.'s are taken into account individually in the estimation of e.s.d.'s in distances, angles and torsion angles; correlations between e.s.d.'s in cell parameters are only used when they are defined by crystal symmetry. An approximate (isotropic) treatment of cell e.s.d.'s is used for estimating e.s.d.'s involving l.s. planes.
Refinement. Refinement of *F*^2^ against ALL reflections. The weighted *R*-factor *wR* and goodness of fit *S* are based on *F*^2^, conventional *R*-factors *R* are based on *F*, with *F* set to zero for negative *F*^2^. The threshold expression of *F*^2^ > σ(*F*^2^) is used only for calculating *R*-factors(gt) *etc*. and is not relevant to the choice of reflections for refinement. *R*-factors based on *F*^2^ are statistically about twice as large as those based on *F*, and *R*- factors based on ALL data will be even larger.

### Fractional atomic coordinates and isotropic or equivalent isotropic displacement parameters (Å^2^)

**Table:**

	*x*	*y*	*z*	*U*_iso_*/*U*_eq_	Occ. (<1)
Cl1	−0.1885 (2)	1.03193 (7)	1.30609 (7)	0.0514 (3)	
Cl2	−0.2647 (2)	0.66171 (7)	1.25574 (6)	0.0410 (3)	
S1	0.00839 (17)	0.62886 (7)	1.07446 (5)	0.0301 (2)	
O1	0.7716 (5)	0.79090 (18)	0.84041 (16)	0.0380 (6)	
N1	0.3542 (6)	0.7790 (2)	0.99367 (18)	0.0301 (6)	
N2	0.3951 (5)	0.7091 (2)	0.94181 (18)	0.0284 (6)	
N3	0.2316 (5)	0.5525 (2)	0.92944 (17)	0.0321 (7)	
N4	0.4208 (5)	0.5638 (2)	0.84780 (18)	0.0330 (7)	
C1	0.1722 (7)	0.9123 (3)	1.1014 (2)	0.0338 (8)	
H1A	0.3000	0.9339	1.0447	0.041*	
C2	0.0985 (8)	0.9801 (3)	1.1546 (2)	0.0376 (8)	
H2A	0.1744	1.0473	1.1351	0.045*	
C3	−0.0885 (7)	0.9488 (3)	1.2373 (2)	0.0360 (8)	
C4	−0.1976 (7)	0.8511 (3)	1.2661 (2)	0.0354 (8)	
H4A	−0.3251	0.8301	1.3230	0.043*	
C5	−0.1205 (7)	0.7840 (3)	1.2119 (2)	0.0296 (7)	
C6	0.0653 (7)	0.8122 (3)	1.1278 (2)	0.0292 (7)	
C7	0.1560 (7)	0.7466 (3)	1.0647 (2)	0.0281 (7)	
C8	0.2259 (7)	0.6250 (3)	0.9714 (2)	0.0294 (7)	
C9	0.6039 (7)	0.7215 (3)	0.8630 (2)	0.0309 (8)	
C10	0.5922 (7)	0.6403 (3)	0.8173 (2)	0.0313 (8)	
C11	0.7890 (7)	0.6472 (3)	0.7261 (2)	0.0371 (9)	
H11A	0.9216	0.5801	0.7374	0.045*	0.509 (8)
H11B	0.9176	0.7053	0.7140	0.045*	0.509 (8)
H11C	0.9466	0.6944	0.7210	0.045*	0.491 (8)
H11D	0.8881	0.5759	0.7305	0.045*	0.491 (8)
C12	0.600 (6)	0.6676 (16)	0.6331 (18)	0.020 (3)	0.509 (8)
C13	0.4824 (18)	0.7651 (7)	0.5769 (5)	0.0307 (18)	0.509 (8)
H13A	0.5246	0.8266	0.5899	0.037*	0.509 (8)
C14	0.302 (4)	0.7744 (17)	0.5012 (10)	0.044 (3)	0.509 (8)
H14A	0.2146	0.8423	0.4631	0.053*	0.509 (8)
C15	0.248 (3)	0.6858 (12)	0.4802 (8)	0.049 (3)	0.509 (8)
H15A	0.1266	0.6927	0.4268	0.059*	0.509 (8)
C16	0.3718 (17)	0.5854 (7)	0.5380 (5)	0.042 (2)	0.509 (8)
H16A	0.3364	0.5239	0.5237	0.051*	0.509 (8)
C17	0.546 (2)	0.5769 (7)	0.6159 (7)	0.034 (2)	0.509 (8)
H17A	0.6267	0.5093	0.6569	0.041*	0.509 (8)
C12X	0.611 (7)	0.6874 (18)	0.6421 (19)	0.023 (3)	0.491 (8)
C13X	0.5639 (18)	0.7976 (7)	0.5985 (6)	0.0300 (18)	0.491 (8)
H13B	0.6560	0.8415	0.6253	0.036*	0.491 (8)
C14X	0.3859 (17)	0.8449 (7)	0.5171 (5)	0.041 (2)	0.491 (8)
H14B	0.3532	0.9199	0.4898	0.050*	0.491 (8)
C15X	0.256 (4)	0.7806 (18)	0.4761 (10)	0.042 (3)	0.491 (8)
H15B	0.1343	0.8127	0.4208	0.050*	0.491 (8)
C16X	0.302 (3)	0.6708 (13)	0.5142 (7)	0.041 (2)	0.491 (8)
H16B	0.2120	0.6277	0.4861	0.049*	0.491 (8)
C17X	0.483 (2)	0.6257 (8)	0.5948 (7)	0.033 (2)	0.491 (8)
H17B	0.5238	0.5507	0.6193	0.040*	0.491 (8)

### Atomic displacement parameters (Å^2^)

**Table:**

	*U*^11^	*U*^22^	*U*^33^	*U*^12^	*U*^13^	*U*^23^
Cl1	0.0638 (7)	0.0300 (5)	0.0509 (6)	0.0054 (5)	0.0255 (5)	−0.0094 (4)
Cl2	0.0471 (5)	0.0458 (6)	0.0308 (5)	−0.0175 (4)	0.0167 (4)	−0.0126 (4)
S1	0.0230 (4)	0.0477 (5)	0.0195 (4)	−0.0078 (4)	0.0029 (3)	−0.0101 (4)
O1	0.0324 (13)	0.0363 (14)	0.0368 (13)	−0.0029 (11)	0.0121 (10)	−0.0035 (11)
N1	0.0250 (14)	0.0336 (16)	0.0261 (14)	0.0025 (12)	0.0024 (11)	−0.0049 (12)
N2	0.0209 (13)	0.0383 (16)	0.0228 (13)	0.0010 (12)	0.0022 (11)	−0.0076 (12)
N3	0.0220 (14)	0.059 (2)	0.0178 (13)	−0.0098 (14)	0.0029 (10)	−0.0146 (13)
N4	0.0189 (13)	0.065 (2)	0.0189 (13)	−0.0113 (14)	0.0041 (10)	−0.0174 (13)
C1	0.0279 (18)	0.0290 (19)	0.0327 (18)	0.0058 (15)	0.0077 (14)	0.0008 (15)
C2	0.038 (2)	0.0248 (18)	0.0365 (19)	0.0046 (16)	0.0101 (16)	0.0027 (15)
C3	0.0323 (19)	0.0298 (19)	0.038 (2)	0.0105 (15)	0.0043 (15)	−0.0056 (15)
C4	0.0338 (19)	0.034 (2)	0.0289 (18)	0.0028 (16)	0.0108 (14)	−0.0020 (15)
C5	0.0270 (17)	0.0313 (18)	0.0253 (16)	−0.0021 (15)	0.0027 (13)	−0.0031 (14)
C6	0.0231 (17)	0.0342 (19)	0.0235 (16)	0.0048 (14)	0.0010 (13)	−0.0036 (14)
C7	0.0210 (16)	0.0343 (19)	0.0219 (16)	0.0001 (14)	−0.0002 (12)	−0.0007 (13)
C8	0.0194 (16)	0.050 (2)	0.0169 (15)	−0.0035 (15)	−0.0006 (12)	−0.0079 (14)
C9	0.0232 (17)	0.040 (2)	0.0215 (16)	0.0038 (16)	0.0003 (13)	−0.0014 (14)
C10	0.0175 (15)	0.054 (2)	0.0195 (15)	−0.0005 (16)	−0.0001 (12)	−0.0087 (15)
C11	0.0227 (17)	0.064 (3)	0.0236 (17)	−0.0066 (17)	0.0083 (13)	−0.0135 (17)
C12	0.019 (5)	0.034 (7)	0.011 (4)	0.005 (5)	0.002 (3)	−0.013 (4)
C13	0.038 (4)	0.035 (4)	0.018 (3)	0.000 (3)	0.008 (3)	−0.010 (3)
C14	0.033 (6)	0.064 (5)	0.020 (6)	0.013 (5)	−0.002 (4)	0.000 (6)
C15	0.033 (5)	0.080 (7)	0.025 (5)	−0.002 (5)	−0.005 (4)	−0.007 (5)
C16	0.048 (4)	0.058 (5)	0.029 (4)	−0.012 (4)	0.013 (3)	−0.027 (4)
C17	0.041 (5)	0.035 (5)	0.024 (4)	−0.004 (4)	0.007 (3)	−0.006 (4)
C12X	0.019 (4)	0.035 (7)	0.016 (6)	−0.008 (5)	0.008 (4)	−0.011 (4)
C13X	0.028 (4)	0.036 (4)	0.022 (4)	0.003 (3)	0.006 (3)	−0.008 (3)
C14X	0.039 (4)	0.051 (5)	0.022 (3)	0.005 (4)	0.009 (3)	0.000 (3)
C15X	0.019 (5)	0.083 (7)	0.014 (6)	0.009 (5)	0.001 (4)	−0.010 (6)
C16X	0.024 (5)	0.074 (6)	0.023 (5)	−0.004 (5)	0.003 (4)	−0.015 (5)
C17X	0.039 (5)	0.042 (5)	0.019 (4)	−0.005 (4)	0.011 (3)	−0.012 (4)

### Geometric parameters (Å, º)

**Table:**

Cl1—C3	1.730 (4)	C11—H11A	0.9900
Cl2—C5	1.741 (3)	C11—H11B	0.9900
S1—C8	1.736 (3)	C11—H11C	0.9900
S1—C7	1.749 (3)	C11—H11D	0.9900
O1—C9	1.216 (4)	C12—C13	1.36 (2)
N1—C7	1.306 (4)	C12—C17	1.375 (18)
N1—N2	1.373 (4)	C13—C14	1.370 (14)
N2—C8	1.371 (4)	C13—H13A	0.9500
N2—C9	1.403 (4)	C14—C15	1.37 (3)
N3—C8	1.305 (4)	C14—H14A	0.9500
N3—N4	1.382 (3)	C15—C16	1.402 (16)
N4—C10	1.299 (4)	C15—H15A	0.9500
C1—C2	1.374 (5)	C16—C17	1.382 (12)
C1—C6	1.406 (4)	C16—H16A	0.9500
C1—H1A	0.9500	C17—H17A	0.9500
C2—C3	1.387 (4)	C12X—C13X	1.40 (2)
C2—H2A	0.9500	C12X—C17X	1.425 (19)
C3—C4	1.379 (5)	C13X—C14X	1.395 (10)
C4—C5	1.380 (4)	C13X—H13B	0.9500
C4—H4A	0.9500	C14X—C15X	1.40 (2)
C5—C6	1.395 (4)	C14X—H14B	0.9500
C6—C7	1.474 (4)	C15X—C16X	1.39 (3)
C9—C10	1.458 (5)	C15X—H15B	0.9500
C10—C11	1.518 (4)	C16X—C17X	1.391 (14)
C11—C12X	1.42 (3)	C16X—H16B	0.9500
C11—C12	1.57 (2)	C17X—H17B	0.9500
			
C8—S1—C7	88.09 (16)	H11A—C11—H11B	107.9
C7—N1—N2	109.0 (3)	C12X—C11—H11C	109.8
C8—N2—N1	117.1 (2)	C10—C11—H11C	109.8
C8—N2—C9	121.1 (3)	C12X—C11—H11D	109.8
N1—N2—C9	121.9 (3)	C10—C11—H11D	109.8
C8—N3—N4	116.4 (3)	H11C—C11—H11D	108.2
C10—N4—N3	121.4 (3)	C13—C12—C17	121.7 (16)
C2—C1—C6	122.4 (3)	C13—C12—C11	124.0 (12)
C2—C1—H1A	118.8	C17—C12—C11	114.2 (16)
C6—C1—H1A	118.8	C12—C13—C14	119.8 (13)
C1—C2—C3	118.8 (3)	C12—C13—H13A	120.1
C1—C2—H2A	120.6	C14—C13—H13A	120.1
C3—C2—H2A	120.6	C13—C14—C15	120.3 (15)
C4—C3—C2	120.7 (3)	C13—C14—H14A	119.9
C4—C3—Cl1	118.9 (3)	C15—C14—H14A	119.9
C2—C3—Cl1	120.4 (3)	C14—C15—C16	119.7 (10)
C3—C4—C5	119.6 (3)	C14—C15—H15A	120.1
C3—C4—H4A	120.2	C16—C15—H15A	120.1
C5—C4—H4A	120.2	C17—C16—C15	119.4 (9)
C4—C5—C6	121.8 (3)	C17—C16—H16A	120.3
C4—C5—Cl2	115.9 (2)	C15—C16—H16A	120.3
C6—C5—Cl2	122.2 (3)	C12—C17—C16	119.1 (12)
C5—C6—C1	116.6 (3)	C12—C17—H17A	120.5
C5—C6—C7	125.7 (3)	C16—C17—H17A	120.5
C1—C6—C7	117.7 (3)	C13X—C12X—C11	117.8 (13)
N1—C7—C6	118.7 (3)	C13X—C12X—C17X	116.3 (19)
N1—C7—S1	116.4 (2)	C11—C12X—C17X	125.9 (17)
C6—C7—S1	124.9 (2)	C14X—C13X—C12X	122.0 (12)
N3—C8—N2	125.2 (3)	C14X—C13X—H13B	119.0
N3—C8—S1	125.4 (2)	C12X—C13X—H13B	119.0
N2—C8—S1	109.4 (2)	C13X—C14X—C15X	119.1 (10)
O1—C9—N2	121.9 (3)	C13X—C14X—H14B	120.4
O1—C9—C10	127.1 (3)	C15X—C14X—H14B	120.4
N2—C9—C10	111.0 (3)	C16X—C15X—C14X	121.5 (11)
N4—C10—C9	124.8 (3)	C16X—C15X—H15B	119.3
N4—C10—C11	117.6 (3)	C14X—C15X—H15B	119.3
C9—C10—C11	117.6 (3)	C15X—C16X—C17X	118.2 (13)
C12X—C11—C10	109.5 (13)	C15X—C16X—H16B	120.9
C10—C11—C12	112.3 (11)	C17X—C16X—H16B	120.9
C10—C11—H11A	109.2	C16X—C17X—C12X	122.7 (14)
C12—C11—H11A	109.2	C16X—C17X—H17B	118.7
C10—C11—H11B	109.2	C12X—C17X—H17B	118.7
C12—C11—H11B	109.2		
			
C7—N1—N2—C8	1.3 (4)	N1—N2—C9—O1	4.5 (5)
C7—N1—N2—C9	−177.7 (3)	C8—N2—C9—C10	4.1 (4)
C8—N3—N4—C10	2.7 (4)	N1—N2—C9—C10	−177.0 (3)
C6—C1—C2—C3	−0.1 (5)	N3—N4—C10—C9	−0.7 (5)
C1—C2—C3—C4	0.4 (5)	N3—N4—C10—C11	−179.1 (3)
C1—C2—C3—Cl1	−179.7 (3)	O1—C9—C10—N4	175.7 (3)
C2—C3—C4—C5	−0.2 (5)	N2—C9—C10—N4	−2.7 (4)
Cl1—C3—C4—C5	179.8 (3)	O1—C9—C10—C11	−5.8 (5)
C3—C4—C5—C6	−0.2 (5)	N2—C9—C10—C11	175.7 (3)
C3—C4—C5—Cl2	178.9 (2)	N4—C10—C11—C12X	75.2 (9)
C4—C5—C6—C1	0.4 (5)	C9—C10—C11—C12X	−103.4 (9)
Cl2—C5—C6—C1	−178.6 (2)	N4—C10—C11—C12	62.9 (9)
C4—C5—C6—C7	−179.1 (3)	C9—C10—C11—C12	−115.7 (8)
Cl2—C5—C6—C7	1.9 (5)	C10—C11—C12—C13	84 (2)
C2—C1—C6—C5	−0.3 (5)	C10—C11—C12—C17	−92.4 (19)
C2—C1—C6—C7	179.3 (3)	C17—C12—C13—C14	0 (3)
N2—N1—C7—C6	−177.3 (3)	C11—C12—C13—C14	−175.2 (18)
N2—N1—C7—S1	0.6 (3)	C12—C13—C14—C15	−2 (3)
C5—C6—C7—N1	−173.5 (3)	C13—C14—C15—C16	1 (3)
C1—C6—C7—N1	7.0 (4)	C14—C15—C16—C17	0.4 (19)
C5—C6—C7—S1	8.8 (5)	C13—C12—C17—C16	1 (3)
C1—C6—C7—S1	−170.7 (2)	C11—C12—C17—C16	177.5 (13)
C8—S1—C7—N1	−1.7 (3)	C15—C16—C17—C12	−1.9 (19)
C8—S1—C7—C6	176.0 (3)	C10—C11—C12X—C13X	87 (2)
N4—N3—C8—N2	−1.2 (5)	C10—C11—C12X—C17X	−95 (2)
N4—N3—C8—S1	−180.0 (2)	C11—C12X—C13X—C14X	−178.3 (14)
N1—N2—C8—N3	178.5 (3)	C17X—C12X—C13X—C14X	4 (3)
C9—N2—C8—N3	−2.5 (5)	C12X—C13X—C14X—C15X	−1.6 (19)
N1—N2—C8—S1	−2.5 (3)	C13X—C14X—C15X—C16X	0 (2)
C9—N2—C8—S1	176.5 (2)	C14X—C15X—C16X—C17X	0 (2)
C7—S1—C8—N3	−178.9 (3)	C15X—C16X—C17X—C12X	3 (2)
C7—S1—C8—N2	2.2 (2)	C13X—C12X—C17X—C16X	−5 (3)
C8—N2—C9—O1	−174.4 (3)	C11—C12X—C17X—C16X	177.7 (18)

### Hydrogen-bond geometry (Å, º)

**Table:**

*D*—H···*A*	*D*—H	H···*A*	*D*···*A*	*D*—H···*A*
C2—H2*A*···O1^i^	0.95	2.36	3.226 (5)	151

Symmetry code: (i) −*x*+1, −*y*+2, −*z*+2.

## References

[bb15] Bernstein, J., Davis, R. E., Shimoni, L. & Chang, N.-L. (1995). *Angew. Chem. Int. Ed. Engl.* **34**, 1555–1573.

[bb1] Bruker (2009). *SADABS*, *APEX2* and *SAINT* Bruker AXS Inc., Madison, Wisconsin, USA.

[bb2] Cosier, J. & Glazer, A. M. (1986). *J. Appl. Cryst.* **19**, 105–107.

[bb3] Eue, L. & Tietz, H. (1970). *Pflanzenshutz-Nachrichten Bayer*, **23**, 208–218.

[bb4] Fun, H.-K., Chantrapromma, S., Chandrakantha, B., Isloor, A. M. & Shetty, P. (2011). *Acta Cryst.* E**67**, o205–o206. 10.1107/S1600536810052505 PMC3050181 21522706

[bb5] Holla, B. S., Kalluraya, B. & Sridhar, K. R. (1988). *Rev. Roum. Chim.* **33**, 277–288.

[bb6] Holla, B. S., Sarojini, B. K. & Gonsalves, R. (1998). *Il Farmaco*, **53**, 395–398. 10.1016/s0014-827x(98)00036-6 9764471

[bb7] Jia, W., Wang, Z., Jia, X., Zhang, J. & Wang, W. (2011). *Acta Cryst.* E**67**, o1093. 10.1107/S1600536811012748 PMC3089198 21754413

[bb8] Kurtzer, F. (1965). *Adv. Heterocycl. Chem.* **5**, 119–204.

[bb9] Ma, W.-W. & Yang, M.-H. (2008). *Acta Cryst.* E**64**, m630.

[bb10] Sandstrom, J. (1968). *Adv. Heterocycl. Chem.* **9**, 165–209. 10.1016/s0065-2725(08)60373-6 4872977

[bb11] Sheldrick, G. M. (2008). *Acta Cryst.* A**64**, 112–122. 10.1107/S0108767307043930 18156677

[bb12] Spek, A. L. (2009). *Acta Cryst.* D**65**, 148–155. 10.1107/S090744490804362X PMC2631630 19171970

[bb13] Yu, M., Zhang, K., Qian, K.-D., Zhang, L.-X. & Liu, Y.-Z. (2007). *Acta Cryst.* E**63**, o2551.

[bb14] Zhang, J., He, Q., Jiang, Q., Mu, H. & Wan, R. (2011). *Acta Cryst.* E**67**, o2255. 10.1107/S1600536811030868 PMC3200604 22058914

